# Procalcitonin and blood lactate level as predictive biomarkers in pediatric multiple trauma patients’ pediatric intensive care outcomes: A retrospective observational study

**DOI:** 10.1097/MD.0000000000036289

**Published:** 2023-12-08

**Authors:** Mustafa Colak, Mehmet Arda Kilinc, Ramazan Güven, Nurettin Onur Kutlu

**Affiliations:** a Department of Paediatric Intensive Care Unit, Basaksehir Cam and Sakura City Hospital, Istanbul, Turkey; b Department of Emergency Medicine, Basaksehir Cam and Sakura City Hospital, Istanbul, Turkey; c Department of Paediatric Intensive Care Unit, Bagcilar Training and Research Hospital, Istanbul, Turkey.

**Keywords:** Lactic acid, multiple trauma, pediatric intensive care unit, procalcitonin

## Abstract

Pediatric trauma represents a significant source of morbidity and mortality in children, encompassing a broad spectrum of injuries. Despite advancements in the treatment and prevention of injuries, the risk of trauma in children remains a persistent concern. Severe trauma cases often necessitate admission to a pediatric intensive care unit (PICU). Procalcitonin, an essential biomarker that elevates bacterial infections and trauma, and elevated lactate levels can signal adverse outcomes in critically ill patients. This study retrospectively examined pediatric patients with multiple trauma treated at the Başakşehir Çam and Sakura City Hospital PICU between 2021 and 2023. The analysis sought to evaluate the relationship between initial procalcitonin and lactate levels with the duration of stay in the PICU, the length of invasive mechanical ventilation (IMV), and the duration of inotropic support. Furthermore, a comparison was made between procalcitonin and lactate levels in survivors and non-survivors, analyzing their potential influence on PICU outcomes and mortality. For pediatric multi-trauma patients, the median duration of stay in the PICU was found to be 3 days. Among these patients, 32% necessitated IMV support and utilized it for a median of 5 days. Additionally, 36% of these patients were provided inotropic drug support for a median time of 6 days. The observed mortality rate was 11%. Procalcitonin and blood lactate levels were found to have significant predictive power for mortality with odds ratios of 1.05 (*P* = .04) and 1.87 (*P* = .02), respectively. Both blood lactate and procalcitonin levels were significantly associated with the duration of IMV support, the period of inotropic drug administration, and the length of PICU stay (*P* < .01; *P* < .01; *P* < .01, respectively). this research underscores the prognostic value of initial procalcitonin and lactate levels about the intensive care trajectory of pediatric trauma patients. The findings suggest that both procalcitonin and lactate levels may play pivotal roles as potential biomarkers in predicting and managing clinical outcomes in this population.

## 1. Introduction

Pediatric trauma is a leading cause of morbidity and mortality among children worldwide. It represents a significant public health challenge due to its high incidence. Moreover, its potential to cause long-term disability places a heavy burden on healthcare services.^[1–3]^ There are various causes of trauma, ranging from falls to traffic accidents, burns, and other accidental injuries.^[4,5]^

Despite advancements in injury care and prevention strategies, the risk of accidents remains a serious threat to children health and quality of life.^[6]^ Severe pediatric trauma cases often require admission to the intensive care unit (ICU), where patients can receive specialized care and continuous monitoring. A pediatric ICU (PICU), supported by a well-equipped and experienced team, is a key element of any pediatric trauma center. PICU possess advanced monitoring devices, equipment, medications, and technologies tailored to meet the needs of pediatric patients.^[7]^

In patients with multiple traumas, brain trauma is a commonly encountered condition. Traumatic brain injury (TBI) is characterized by increased inflammatory biomarkers mediated by the immune system. After TBI, the activation of microglial cells and the accumulation of T cells in the blood-brain barrier trigger the production of oxidative stress and pro-inflammatory mediators.

Immediately following brain injury, pro-inflammatory cytokines such as interleukin-1β, interleukin-6, and tumor necrosis factor-alpha are produced in high amounts, along with transforming growth factor-beta. These factors exacerbate trauma by increasing oxidative stress and matrix metalloproteinases, thereby delaying the healing process. This inflammation leads to immune system dysfunctions such as systemic inflammatory response syndrome and compensatory anti-inflammatory response syndrome. As a result, organ dysfunctions and infections arise.^[8,9]^

For patients in ICU, nutrition and complementary therapies can be used to reduce oxidative stress and inflammation. Studies have indicated that using French maritime pine bark extract (Oligopin) can enhance recovery in severely injured TBI patients in the ICU, leading to higher survival rates. Furthermore, combining Propolis and melatonin might decrease infection, inflammation, and oxidative stress levels. This combination could potentially reduce the length of hospital stay and improve survival rates for ICU patients.^[8,10,11]^

Procalcitonin is recognized as a biomarker commonly used in the diagnosis of bacterial infections and sepsis. It appears that procalcitonin can be used effectively as a sepsis marker in burn patients. This molecule serves as the precursor to the calcitonin hormone. Its serum levels rise not only in response to microbial infections but also in patients with severe trauma.^[12–14]^ Elevated lactate levels have been associated with mortality in critically ill children.^[15]^ Various scoring systems are used to assess the severity of trauma.^[16]^ However, current scoring systems often do not include potentially critical factors such as procalcitonin and elevated lactate levels.

The high preventable morbidity and mortality following pediatric trauma is a critical issue for public health. Although previous studies have examined the relationship between trauma severity and procalcitonin levels in adults, the nature of this relationship in pediatric patients has not yet been definitively determined. Furthermore, although research shows the importance of blood lactate levels in critically ill children, the relationship between trauma severity and elevated blood lactate levels has not been established. This resource aims to evaluate the potential impact of procalcitonin levels and elevated lactate levels on mortality and PICU outcomes in children with severe trauma as different from previous studies.

## 2. Materials and methods

### 2.1. Design

This study used a retrospective observational design and was conducted in the PICU of Başakşehir Çam and Sakura City Hospital. The PICU is a tertiary intensive care unit that provides 16 beds and treats an average of 550 patients yearly.

The study included patients who required treatment in the PICU after multiple trauma. Demographic characteristics and trauma etiologies of the patients were assessed. Radiological images and their reports after trauma were examined. After admission to the PICU, vital signs and laboratory values were evaluated. In addition to this clinical and radiological information, the supportive treatments applied in the PICU and the duration of these treatments were analyzed. The length of stay of the patients in the PICU was recorded.

### 2.2. Ethical statement

The study was approved by Başakşehir Çam and Sakura City Hospital ethics committee with number 2023.06.244.

### 2.3. Patient selection and data collection

Patients admitted to the Başakşehir Çam and Sakura City Hospital PICU between January 1, 2021, and August 1, 2023, were retrospectively screened. During this period, the medical records of a total of 1668 patients treated in the unit were analyzed to identify patients with multiple trauma by chart review.

Among the examined patients, those admitted to intensive care due to multiple trauma were identified. However, patients admitted due to burns, near-drowning incidents, or suicidal hangings were excluded from the study. After these exclusion criteria, 154 patients were included in the analysis.

Demographic and clinical data related to the patients were accessed retrospectively from the hospital information system.

After admission to the PICU, patients’ Glasgow coma scores, pediatric trauma scores, duration of intensive care and hospital stay, inotropic drug support, invasive mechanical ventilator support duration, initial vital signs, initial blood gas, and blood biochemistry values were recorded. Culture samples were taken within the first 48 hours and their results were also included in the records.

### 2.4. Statistical analysis

SPSS version 20 (IBM Corp., Armonk, NY) was used for data analysis. The normal distribution of data was assessed with the Shapiro–Wilk test. Patients’ age distributions are presented as median and range values. Glasgow coma score, pediatric trauma score, vital signs, laboratory blood values, duration in pediatric intensive care, duration of hospital stay, duration of invasive mechanical ventilator support, and duration of inotropic drug support are expressed as median, first (Q1), and third (Q3) quartiles. Differences in laboratory values between deceased and surviving patients, differences between those requiring and not requiring invasive mechanical ventilation (IMV), and differences between patients receiving and not receiving inotropic drug support were analyzed with the Mann–Whitney *U* test. The relationship between procalcitonin and blood gas parameters with the duration of invasive mechanical ventilator support, duration of inotropic drug use, time spent in the PICU, and total hospital stay duration was examined with the Spearman correlation test. The influence of procalcitonin, alongside blood gas parameters including pH, lactate, sodium bicarbonate (NaHCO_3_), and base excess on mortality, was evaluated using logistic regression analysis. To assess their impact on the duration of stay in the PICU, a multivariate linear regression analysis was employed. In statistical analyses, a *P* value of < 0.05 was considered statistically significant.

## 3. Results

Of the 154 patients included in this study, 95 were male and 59 were female. The median age of the patients was 63 months (range: 3–212 months). The distribution according to the cause of trauma was as follows: 77 falls, 45 out-of-vehicle traffic accidents, 18 in-vehicle traffic accidents, and 14 other causes. Among the patients monitored in the PICU, head trauma was the most common injury, followed by lung injuries (Table 1).

**Table 1:** Distribution of injuries.

Head injuries	Number (%)
Cranial fracture	115 (74.7%)
Hemorrhage	93 (60.4%)
Edema	20 (13%)
Lung injuries	
Pneumothorax	78 (50.6%)
Hemothorax	15 (9.7%)
Contusion	95 (61.7%)
Abdominal injuries	
Liver	34 (22.1%)
Spleen	20 (13%)
Kidney	17 (11%)
Other	23 (14.9%)
Extremity fracture	59 (38.3%)
Spinal injury	6 (3.9%)
Pelvic fracture	20 (13%)

At the time of PICU admission, the median Glasgow Coma Score was 11 (Q25–Q75: 6–15). The median pediatric trauma score was determined as 4 (Q25–Q75: 1–8). After admission, vital signs showed a median systolic blood pressure of 110 mm Hg (Q25–Q75: 103–118 mm Hg), median diastolic blood pressure of 66 mm Hg (Q25–Q75: 60–74 mm Hg), median heart rate of 122 beats per minute (Q25–Q75: 110–135 bpm), median respiratory rate of 25 breaths per minute (Q25–Q75: 21–29), and median peripheral oxygen saturation of 95% (Q25–Q75: 91%–99%). Blood values obtained upon admission are detailed in Table 2.

**Table 2:** Laboratory values at admission to the pediatric intensive care unit.

	All	Survivors	Non-survivors	*P* value
Blood gas				
pH	7.31(7.23–7.3)	7.33(7.26–7.4)	7.2(7.13–7.25)	<0.01
PCO_2_	41.4(34–44)	42(36–46)	38(32–43)	0.48
iCa	1.24 (1.2–1.4)	1.14 (1.1–1.19)	1.2 (1.1–1.25)	0.11
Lactate	2.7(1.3–4.6)	2.2(1.3–3.9)	7.5 (6.1–7.9)	<0.01
Base Excess	−4.4 {(−7.9)–(−2)}	−3 {(−6.5)–(−1.3)}	−12.6 {(−18.7)–(−9.8)}	<0.01
NaHCO_3_	21 (18.9–23)	21.6 (19.7–23.1)	13.4 (10.5–17.8)	<0.01
WBC	15.9(13–20.2)	16.1(13.2–20.2)	12.4 (10.2–17.8)	0.32
Hb	11(9.6–11.9)	11(9.7–12)	9.8(7.2–10.4)	0.04
PLT	298(237–385)	315(265–387)	137(62–182)	<0.01
APTT	28.9(25.7–33)	28(25.3–31.1)	105(68–114)	<0.01
INR	1.2(1.1–1.4)	1.18(1−1.34)	2.46(1.68–2.67)	<0.01
Glucose	156(128–190)	158(130–189)	129(94–164)	0.58
BUN	27.2(22–38.1)	25(21.8–35)	37.4(23.7–50.2)	0.12
Creatinine	0.46(0.32–0.7)	0.42(0.29–0.66)	0.70(0.6–0.95)	<0.01
ALT	56(24–230)	42(21–211)	160(96–621)	0.04
AST	118(43–447)	108(41–380)	390(227–690)	0.03
CK	647(262–1102)	558(259–1004)	2576(1992–3160)	0.08
Amylase	63(51–103)	61(51–100)	159(102–210)	0.28
Lipase	32(16–71)	31(15–66)	97(81–487)	0.06
CRP	67(23–129)	59(15–87)	89 (36–134)	0.46
Procalcitonin	4.2(0.7–11.2)	4(0.6–9.8)	21.6 (7.8–58)	<0.01

Values are presented as median (25th quartile–75th quartile). The Mann–Whitney *U* test was used for statistical analysis. *P* < .05 was considered statistically significant.

ALT = Alanine aminotransferase, APTT = activated partial thromboplastin time, AST = aspartate aminotransferase, BUN = blood urea nitrogen, CK = creatine kinase, CRP = C-reactive protein, Hb = hemoglobin, iCa = ionized calcium, INR = international normalized ratio, NaHCO_3_ = sodium bicarbonate, PCO_2_ = partial pressure of carbon dioxide, PLT = platelets, WBC = white blood cells.

Patients’ median stay duration in the PICU was 3 days (Q25–Q75: 2–9 days). The median general hospital stay was 8 days (Q25–Q75: 5–14 days). 32% (n = 49) of patients treated in the PICU required IMV support. For these patients, the median duration of IMV use was 5 days (Q25–Q75: 2–9 days). 36% (n = 56) of the participant patients received inotropic drug support, with a median duration of 6 days (Q25–Q75: 2–10 days). As a result of the study, 11% (n = 17) of monitored patients passed away.

**Figure 1.**
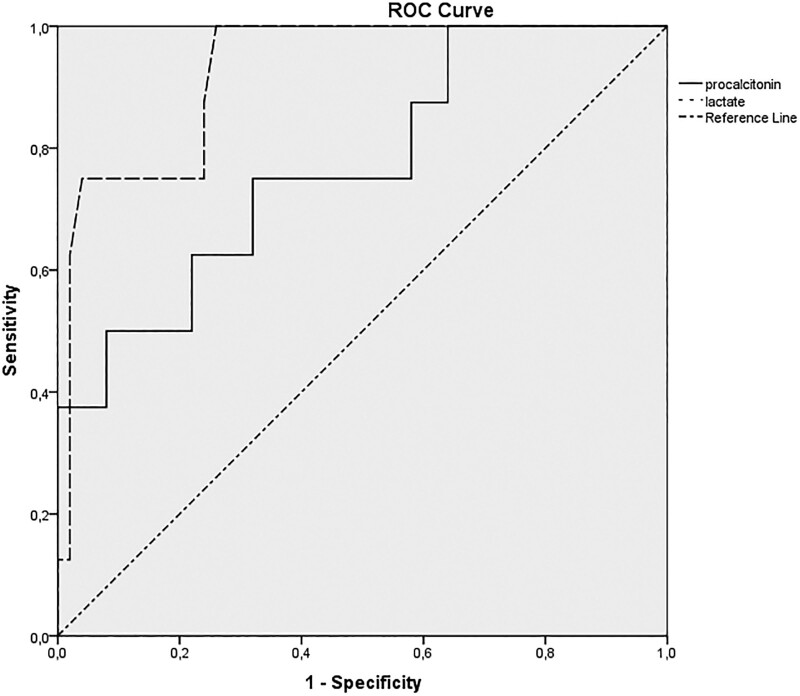
ROC analysis of procalscitonin anad lactete for mortality. ROC = receiver operating characteristic.

A logistic regression analysis was conducted to shed light on the determinants of mortality. The analysis indicated that the influence of procalcitonin levels on mortality yielded an odds ratio of 1.05. This ratio 95% confidence interval (CI) is anchored between 1.01 and 1.08, signifying statistical significance with a *P* value of .045. When assessing the role of blood lactate levels on mortality, the odds ratio stood at 1.87. Its 95% CI lies between 0.99 and 3.9, presenting a statistically significant outcome as indicated by a *P* value of .02 (Table 3). In predicting mortality, the receiver operating characteristic curve revealed an area under the curve of 0.709 for procalcitonin, while lactate demonstrated a notably higher area under the curve of 0.947 (Fig. 1).

**Table 3:** Mortality prediction factors using logistic regression analysis.

	Odds ratio	*P* value	95% CI interval
Procalcitonin	1.05	0.04	1.01–1.08
pH	1.2	0.03	0.9–1.62
Lactate	1.87	0.02	0.99–3.9
Base excess	0.78	0.17	0.54–1.12
NaHCO_3_	0.64	0.21	0.49–0.83

Nagelkarke R²: 0.63, X² (Chi-square): 22.5. A *P* value of < 0.05 is considered statistically significant.

NaHCO_3_ = sodium bicarbonate, pH = blood gas pH.

When the procalcitonin levels were compared between patients with and without IMV support, the median procalcitonin value was 9.58 for patients with IMV support and 1.5 for patients without IMV support (*P* < .01). Similarly, procalcitonin values in patients requiring inotropic support were higher compared to others, with median values of 10.4 and 1.9, respectively (*P* < .01). The median lactate value for supported patients was 4.5, while for non-supported patients, it was 1.6 (*P* < .01). The median lactate levels for patients needing inotropic support were 5.3, whereas for those not needing inotropic support, it was 1.3 (*P* < .01).

Blood gas analysis results concerning blood pH, lactate, base deficit, NaHCO_3_, C-reactive protein (CRP), and procalcitonin parameters were examined in detail for potential relationships with length of IMV support, length of inotropic drug support, and length of stay in PICU. The analysis determined a statistically significant and positive correlation between procalcitonin levels and the length of IMV support, length of inotropic drug support, and length of stay in PICU. Additionally, a notable correlation between lactate levels from the blood gas analysis and the durations of IMV support and inotropic drug support was established (Table 4). A Multivariate Linear Regression Analysis was conducted for a detailed analysis of factors affecting the PICU stay duration. As a result, procalcitonin levels have a significant coefficient on stay duration, specifically, a coefficient value of 0.25. The determined 95% CI for this coefficient ranges from 0.1 to 0.33, and the obtained *P* value < 0.01 indicates that this relationship is statistically significant. Within the same analysis, the effect of lactate was also evaluated, and its coefficient was determined to be 1.22, with a significance level of *P* = .02. On the other hand, the effects of blood pH, base deficit, and NaHCO_3_ on the length of stay in PICU were found to be statistically insignificant (Table 5).

**Table 4:** Correlation of laboratory parameters with PICU course and length of stay.

	Procalcitonin	CRP	pH	Lactate	Base Excess	NaHCO_3_
Length of stay in PICU	rho: 0.69	rho: 0.22	rho: 0.43	rho: 0.31	rho: −0.39	Rho: −0.29
	*P* < 0.01	p:0.2	*P* < 0.01	*P* < 0.01	*P* < 0.01	*P* < 0.01
Length of IMV support	rho: 0.51	rho: 0.17	rho: 0.582	rho: 0.54	rho: 0.43	rho: 0.37
	*P* < 0.01	p:0.38	*P* < 0.01	*P* < 0.01	*P* < 0.01	*P* < 0.01
Length of inotrope therapy	rho: 0.43	rho: 0.15	rho: 0.532	rho: 0.56	rho: 0.39	rho: 0.35
	*P* < 0.01	p:0.43	*P* < 0.01	*P* < 0.01	*P* < 0.01	*P* < 0.01

Correlations in the table were analyzed using the Spearman correlation test. *P* < .05 was considered statistically significant.

CRP = C-reactive protein, IMV = invasive mechanical ventilation, NaHCO_3_ = sodium bicarbonate, pH = blood gas pH, PICU = pediatric intensive care unit.

**Table 5:** Predictors of length of stay in pediatric intensive care unit using multivariate linear regression analysis.

	Coefficents	Standart error	t	p	95% CI interval
Procalcitonin	0.25	0.06	3.98	<0.01	0.1–0.33
pH	−25.9	18.7	−1.3	0.174	(−35.1)–(−13.4)
Lactate	1.22	0.49	2.1	0.02	0.5–2
Base excess	−0.9	0.53	−1.6	0.87	(−1.9)–(0.17)
NaHCO_3_	−0.2	0.58	−0.76	0.79	(−0.73)–(0.4)

*P* < .05 was considered statistically significant.

NaHCO_3_ = sodium bicarbonate, pH = blood gas pH.

The initial CRP and procalcitonin values of the patients were compared with the culture results showing microbial growth within the first 48 hours. The median procalcitonin value was 3.3 (Q25–Q75: 0.6–9.7) in patients without microbial growth and 7.2 (Q25–Q75: 8–56.2) in those with growth. For CRP values, the median value was 55 (Q25–Q75: 8–96) in the group without growth and 76 (Q25–Q75: 58–153) in the group with growth. No statistically significant difference was found between these 2 groups in terms of procalcitonin and CRP values (respectively, p:0.41 and p:0.17).

## 4. Discussion

This study aimed to evaluate the relationship between blood procalcitonin and blood lactate levels with prognosis in patients admitted to the PICU following trauma. The most critical finding revealed a statistically significant correlation between procalcitonin levels at the initial moment of PICU admission and mortality. According to this result, procalcitonin might be a potential biomarker for predicting post-traumatic mortality.

In pediatric patients, the relationship between post-traumatic procalcitonin and sepsis and microbiological culture positivity in the PICU has been extensively investigated in the literature.^[17–19]^ There are also studies showing that it can be used for sepsis detection in critically ill burn patients.^[14]^ However, studies in adult populations have also examined the relationship between trauma severity and procalcitonin levels.^[12,20]^ Our study is among the rare works confirming a similar association in the pediatric population.

Statistically significant differences were detected between the procalcitonin levels of survivors and non-survivors. As per these findings, procalcitonin could be a valuable biomarker in assessing post-traumatic mortality risk. A notable relationship was also identified between procalcitonin levels and length of stay in PICU and hospital, indicating its potential utility in predicting patient treatment and recovery processes.

Patients receiving invasive mechanical ventilatory support had significantly higher procalcitonin levels compared to those without such support. Similarly, those needing inotropic support also had statistically elevated procalcitonin levels. Procalcitonin levels correlated positively with the duration of both IMV and inotropic support. These findings suggest a direct association between the severity of trauma and procalcitonin levels, illuminating its relationship with length of stay in PICU and hospital. Patients requiring more intensive organ support tend to stay longer in the PICU and require prolonged rehabilitation post-discharge.

After TBI, proinflammatory cytokines are produced in large amounts. These worsen the trauma and are known to delay healing by producing oxidative stress and matrix metalloproteinases. Similar to this situation, we think that increased procalcitonin may be a biomarker of severe screening and long-lasting supportive treatments.^[8,9]^

Procalcitonin is a widely used marker for diagnosing infection. Previous literature has noted its use in diagnosing sepsis in multiple trauma patients.^[21,22]^ However, our study approach focused solely on evaluating procalcitonin values at admission. While higher procalcitonin levels were observed in patients with positive culture results, this discrepancy wasn’t statistically significant. This may stem from our study focus only on initial procalcitonin values. Furthermore, this elevation in procalcitonin levels suggests trauma as a possible primary cause.

However, no statistical correlation was observed between CRP values and clinical indicators like mortality and length of stay in PICU. It is known that the procalcitonin response emerges much quicker post multiple trauma compared to CRP.^[23]^ In our study, we concentrated solely on the CRP values of the initial blood samples taken upon PICU admission. Therefore, the absence of a distinct relationship during this early phase, when the CRP response may not have fully developed, is likely.

Blood gas analysis results at PICU admission, particularly concerning lactate level, pH value, base deficit, and NaHCO_3_ levels, are critically important. Comparing these values between survivors and non-survivors revealed statistically significant differences. Additionally, a pronounced correlation was found between these values and length of stay in PICU, length of IMV support, and length of inotropic drug support. This association with mortality is already known in existing literature.^[24]^ Their relationship with IMV support duration, inotropic drug support duration, and PICU stay in pediatric patients presents a novel contribution to the literature through this study.

Lactate levels rise as an indicator of tissue hypoxia, and elevated lactate levels indicate a disruption in cellular metabolism and an increased risk of organ dysfunction.^[25]^ In critically ill children, elevated lactate at hospital admission and lactate clearance are associated with mortality.^[26,27]^ This study confirms this in a specific patient group admitted to the ICU post-multiple trauma. Our findings confirm that high lactate levels are associated with adverse clinical outcomes and extended ICU stays.

Based on our findings, initial procalcitonin and lactate levels in multiple trauma patients admitted to the PICU provide crucial information about patient prognosis. However, several limitations must be considered when interpreting these results. This study is a single-center retrospective investigation, limiting the generalizability of its outcomes. Furthermore, due to patients having suffered multiple trauma, interactions between head, thoracic, and abdominal injuries might exist. This complicates determining which trauma type (head, thorax, or abdomen) has a more dominant influence on outcomes. Another limitation is the potential interventions patients might have received before ICU admission, which weren’t evaluated in our study. These factors could be decisive for patient prognosis, and this gap should be considered in interpreting our findings.

Given our results, we can offer some directions for future research. Firstly, since our study is single-centered, multi-center studies across various geographical areas and health systems are recommended. As our study encompasses a broad spectrum of multiple trauma patients, the effects of specific injury types need more detailed examination. Also, the lack of detailed evaluation of pre-ICU interventions means we might have overlooked the potential impacts of these variables. Finally, investigating other biomarkers potentially playing a role in post-traumatic prognosis could provide a more comprehensive understanding of this domain. Considering these suggestions for future studies can contribute to a more holistic approach to trauma care.

## 5. Conclusion

This study investigated the association between procalcitonin and blood lactate levels at the time of admission and the intensive care process in pediatric patients with multiple trauma. The findings revealed a significant relationship between these biomarkers and the length of stay in PICU, length of IMV support, and length of inotropic support. These results suggest that procalcitonin and blood lactate levels might be valuable in predicting the clinical course of pediatric trauma patients. Consequently, it is inferred that these metrics could serve as significant biomarkers in assessing the risk of complications following trauma in this population.

## Author contributions

**Conceptualization:** Mustafa Colak, Ramazan Guven.

**Data curation:** Mehmet Arda Kilinc.

**Investigation:** Mustafa Colak.

**Methodology:** Mustafa Colak, Ramazan Guven.

**Resources:** Mehmet Arda Kilinc, Nurettin Onur Kutlu.

**Software:** Ramazan Guven.

**Supervision:** Nurettin Onur Kutlu.

**Writing – original draft:** Mustafa Colak.

**Writing – review & editing:** Mustafa Colak.
